# 2-(1,3-Benzothia­zol-2-yl)guanidine

**DOI:** 10.1107/S160053681100732X

**Published:** 2011-03-05

**Authors:** Shaaban Kamel Mohamed, Mahmoud A. A. El-Remaily, Ahmed M. Soliman, Atash V. Gurbanov, Seik Weng Ng

**Affiliations:** a Chemistry & Environmental Science Division, School of Science, Manchester Metropolitan University, England; b Department of Chemistry, Sohag University, Sohag, Egypt; c Department of Organic Chemistry, Baku State University, Baku, Azerbaijan; d Department of Chemistry, University of Malaya, 50603 Kuala Lumpur, Malaysia

## Abstract

In the title comound, C_8_H_8_N_4_S, one of the two independent mol­ecules is essentially planar (r.m.s. deviation = 0.025 Å), while the other is slightly buckled (r.m.s. deviation = 0.131 Å) with the guanidine unit bent out of the plane of the fused-ring system by 16.8 (1)°. In the crystal, inter­molecular N—H⋯N hydrogen bonds between the two independent mol­ecules give rise to a hydrogen-bonded dimer. Addtional weak inter­molecular N—H⋯N hydrogen bonds connect these dimers into chains along [010]. An intra­molecular N—H⋯N hydrogen bond is also observed in each independent mol­ecule.

## Related literature

For the synthesis, see: Dolzhenko *et al.* (2006 ▶).
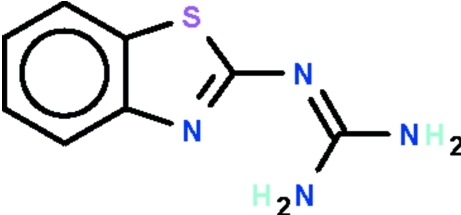


## Experimental

### 

#### Crystal data

C_8_H_8_N_4_S
*M* *_r_* = 192.24
Orthorhombic, 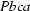

*a* = 10.2970 (3) Å
*b* = 10.0817 (3) Å
*c* = 33.5158 (11) Å
*V* = 3479.32 (18) Å^3^
*Z* = 16
Mo *K*α radiation
μ = 0.33 mm^−1^
*T* = 295 K
0.30 × 0.30 × 0.30 mm

#### Data collection

Bruker APEXII diffractometer
35704 measured reflections
3996 independent reflections
3345 reflections with *I* > 2σ(*I*)
*R*_int_ = 0.026

#### Refinement

*R*[*F* ^2^ > 2σ(*F* ^2^)] = 0.039
*wR*(*F* ^2^) = 0.111
*S* = 1.13
3996 reflections
267 parameters
8 restraints
H atoms treated by a mixture of independent and constrained refinement
Δρ_max_ = 0.22 e Å^−3^
Δρ_min_ = −0.28 e Å^−3^

### 

Data collection: *APEX2* (Bruker, 2005 ▶); cell refinement: *SAINT* (Bruker, 2005 ▶); data reduction: *SAINT*; program(s) used to solve structure: *SHELXS97* (Sheldrick, 2008 ▶); program(s) used to refine structure: *SHELXL97* (Sheldrick, 2008 ▶); molecular graphics: *X-SEED* (Barbour, 2001 ▶); software used to prepare material for publication: *publCIF* (Westrip, 2010 ▶).

## Supplementary Material

Crystal structure: contains datablocks global, I. DOI: 10.1107/S160053681100732X/lh5214sup1.cif

Structure factors: contains datablocks I. DOI: 10.1107/S160053681100732X/lh5214Isup2.hkl

Additional supplementary materials:  crystallographic information; 3D view; checkCIF report

## Figures and Tables

**Table 1:** Hydrogen-bond geometry (Å, °)

*D*—H⋯*A*	*D*—H	H⋯*A*	*D*⋯*A*	*D*—H⋯*A*
N3—H31⋯N1	0.86 (1)	2.00 (2)	2.679 (2)	134 (2)
N3—H32⋯N2^i^	0.86 (1)	2.40 (2)	3.228 (2)	161 (2)
N4—H41⋯N6	0.86 (1)	2.25 (1)	3.084 (3)	165 (3)
N4—H42⋯N8^i^	0.86 (1)	2.50 (2)	3.350 (3)	176 (2)
N7—H71⋯N5	0.86 (1)	2.03 (2)	2.717 (3)	136 (2)
N8—H81⋯N2	0.86 (1)	2.24 (1)	3.096 (3)	177 (2)

Symmetry code: (i) 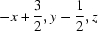.

**Fig. 1.**
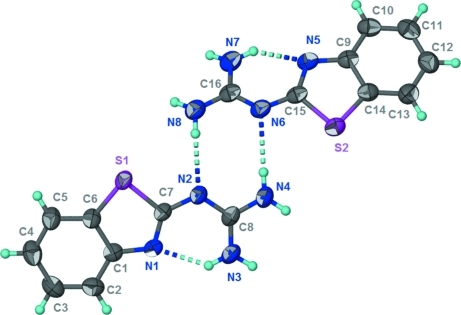
Thermal ellipsoid plot (Barbour, 2001) of the two independent molecules of C8H8N4 with displacement ellipsoids drawn at the 50% probability level. Hydrogen atoms are drawn as spheres of arbitrary radius. Hydrogen bonds are denoted by dashed lines.

## supplementary crystallographic information

### Comment

The title compound was synthesized as an intermediate for the synthesis of other heterocyclic compounds (Dolzhenko *et al.*, 2006). In the title comound, C_8_H_8_N_4_, one of the two independent molecules of is essentially planar (r.m.s. deviation 0.025 Å) while the other is slightly buckled (r.m.s. deviation 0.131 Å) with the guanidine unit bent out of the plane of the fused-ring system by 16.8 (1) °. In the crystal a pair of intermolecular N-H···N hydrogen bonds between the two independent molecules give rise to a hydrogen-bonded dimer (Fig. 1). Addtional weak intermolecular N-H···N hydrogen bonds connect these dimers into one-dimensional chains along [010].

### Experimental

2-Aminothiophenol (0.050 mol) was dissolved in 10% sulfuric acid (50 ml) and to the solution was added cyanoduanidine (0.075 mol). The mixture was heated to give a clear solution. To the cool solution was added 50% sodium hydroxide (10 mol) to precipitate the product. X-ray quality crystals were recrystallized from ethanol in 90% yield. The synthesis was based on a reported procedure (Dolzhenko *et al.*, 2006).

### Refinement

Carbon-bound H-atoms were placed in calculated positions [C–H 0.93 Å; *U*_iso_(H) 1.2*U*_eq_(C)] and were included in the refinement in a riding-model approximation. The amino H-atoms were located in a difference Fourier map, and were refined with a distance restraint of N–H 0.86±0.01 Å; the U_iso_(H) values were refined.

### Crystal data

**Table:**

C_8_H_8_N_4_S	*F*(000) = 1600
*M**_r_* = 192.24	*D*_x_ = 1.468 Mg m^−^^3^
Orthorhombic, *P**b**c**a*	Mo *K*α radiation, λ = 0.71073 Å
Hall symbol: -P 2ac 2ab	Cell parameters from 9882 reflections
*a* = 10.2970 (3) Å	θ = 2.3–27.7°
*b* = 10.0817 (3) Å	µ = 0.33 mm^−^^1^
*c* = 33.5158 (11) Å	*T* = 295 K
*V* = 3479.32 (18) Å^3^	Prism, colorless
*Z* = 16	0.30 × 0.30 × 0.30 mm

### Data collection

**Table:**

Bruker APEXII diffractometer	3345 reflections with *I* > 2σ(*I*)
Radiation source: fine-focus sealed tube	*R*_int_ = 0.026
graphite	θ_max_ = 27.5°, θ_min_ = 2.3°
φ and ω scans	*h* = −13→13
35704 measured reflections	*k* = −13→13
3996 independent reflections	*l* = −43→43

### Refinement

**Table:**

Refinement on *F*^2^	Primary atom site location: structure-invariant direct methods
Least-squares matrix: full	Secondary atom site location: difference Fourier map
*R*[*F*^2^ > 2σ(*F*^2^)] = 0.039	Hydrogen site location: inferred from neighbouring sites
*wR*(*F*^2^) = 0.111	H atoms treated by a mixture of independent and constrained refinement
*S* = 1.13	*w* = 1/[σ^2^(*F*_o_^2^) + (0.0453*P*)^2^ + 1.2618*P*] where *P* = (*F*_o_^2^ + 2*F*_c_^2^)/3
3996 reflections	(Δ/σ)_max_ = 0.001
267 parameters	Δρ_max_ = 0.22 e Å^−^^3^
8 restraints	Δρ_min_ = −0.28 e Å^−^^3^

### Fractional atomic coordinates and isotropic or equivalent isotropic displacement parameters (Å^2^)

**Table:**

	*x*	*y*	*z*	*U*_iso_*/*U*_eq_	
S1	0.48382 (5)	0.66100 (5)	0.659730 (17)	0.05839 (16)	
S2	0.84372 (5)	0.47427 (6)	0.472576 (17)	0.06418 (17)	
N1	0.55487 (14)	0.42140 (14)	0.67760 (4)	0.0474 (3)	
N2	0.64035 (14)	0.51382 (15)	0.61637 (5)	0.0485 (3)	
N3	0.71411 (18)	0.29516 (18)	0.62708 (6)	0.0605 (4)	
H31	0.6709 (19)	0.293 (2)	0.6491 (4)	0.064 (7)*	
H32	0.756 (2)	0.2272 (16)	0.6184 (7)	0.073 (7)*	
N4	0.77196 (19)	0.4128 (2)	0.57119 (5)	0.0649 (5)	
H41	0.753 (3)	0.4781 (19)	0.5560 (6)	0.087 (9)*	
H42	0.8259 (19)	0.3507 (18)	0.5657 (7)	0.071 (7)*	
N5	0.67826 (16)	0.63000 (15)	0.43811 (5)	0.0537 (4)	
N6	0.66455 (16)	0.61327 (15)	0.51004 (5)	0.0534 (4)	
N7	0.47131 (19)	0.70197 (19)	0.48350 (6)	0.0612 (4)	
H71	0.505 (2)	0.692 (3)	0.4602 (4)	0.079 (9)*	
H72	0.3919 (12)	0.722 (2)	0.4882 (7)	0.074 (7)*	
N8	0.5036 (2)	0.68148 (19)	0.55135 (6)	0.0649 (5)	
H81	0.539 (2)	0.635 (2)	0.5699 (5)	0.066 (7)*	
H82	0.4247 (12)	0.706 (3)	0.5554 (7)	0.082 (8)*	
C1	0.47271 (17)	0.46403 (18)	0.70777 (6)	0.0477 (4)	
C2	0.4344 (2)	0.3899 (2)	0.74069 (6)	0.0609 (5)	
H2	0.4661	0.3043	0.7443	0.073*	
C3	0.3497 (2)	0.4436 (3)	0.76785 (7)	0.0682 (6)	
H3	0.3247	0.3939	0.7899	0.082*	
C4	0.3009 (2)	0.5704 (2)	0.76293 (7)	0.0690 (6)	
H4	0.2434	0.6047	0.7817	0.083*	
C5	0.3367 (2)	0.6461 (2)	0.73061 (7)	0.0626 (5)	
H5	0.3037	0.7312	0.7272	0.075*	
C6	0.42309 (18)	0.59256 (18)	0.70318 (6)	0.0504 (4)	
C7	0.56907 (17)	0.51333 (16)	0.65055 (5)	0.0448 (4)	
C8	0.70785 (17)	0.40623 (18)	0.60589 (6)	0.0488 (4)	
C9	0.75591 (18)	0.57694 (17)	0.40817 (5)	0.0495 (4)	
C10	0.7460 (2)	0.6048 (2)	0.36749 (6)	0.0604 (5)	
H10	0.6833	0.6637	0.3582	0.072*	
C11	0.8292 (2)	0.5445 (2)	0.34137 (7)	0.0640 (6)	
H11	0.8221	0.5624	0.3142	0.077*	
C12	0.9238 (2)	0.4574 (2)	0.35448 (7)	0.0685 (6)	
H12	0.9791	0.4175	0.3361	0.082*	
C13	0.9367 (2)	0.4293 (2)	0.39439 (7)	0.0697 (6)	
H13	1.0006	0.3712	0.4033	0.084*	
C14	0.85219 (19)	0.48948 (19)	0.42113 (6)	0.0545 (5)	
C15	0.71308 (18)	0.58623 (17)	0.47312 (6)	0.0494 (4)	
C16	0.5469 (2)	0.66392 (17)	0.51381 (6)	0.0529 (4)	

### Atomic displacement parameters (Å^2^)

**Table:**

	*U*^11^	*U*^22^	*U*^33^	*U*^12^	*U*^13^	*U*^23^
S1	0.0643 (3)	0.0367 (2)	0.0742 (3)	0.0069 (2)	0.0233 (2)	0.0041 (2)
S2	0.0636 (3)	0.0691 (3)	0.0598 (3)	0.0202 (3)	−0.0057 (2)	0.0084 (2)
N1	0.0456 (8)	0.0428 (7)	0.0537 (8)	0.0032 (6)	−0.0053 (6)	0.0014 (6)
N2	0.0488 (8)	0.0429 (8)	0.0537 (8)	0.0055 (6)	0.0022 (7)	−0.0031 (6)
N3	0.0647 (11)	0.0507 (9)	0.0660 (11)	0.0193 (8)	−0.0024 (9)	−0.0044 (8)
N4	0.0678 (11)	0.0718 (12)	0.0551 (10)	0.0255 (10)	0.0021 (8)	−0.0080 (9)
N5	0.0621 (9)	0.0436 (8)	0.0554 (9)	0.0028 (7)	−0.0127 (7)	−0.0002 (7)
N6	0.0627 (10)	0.0432 (8)	0.0544 (9)	0.0061 (7)	−0.0108 (7)	−0.0043 (7)
N7	0.0544 (10)	0.0582 (10)	0.0710 (12)	0.0035 (8)	−0.0113 (9)	−0.0030 (9)
N8	0.0742 (13)	0.0538 (10)	0.0667 (12)	0.0118 (9)	−0.0006 (10)	−0.0019 (8)
C1	0.0418 (9)	0.0473 (9)	0.0539 (10)	−0.0037 (7)	−0.0055 (7)	−0.0004 (8)
C2	0.0602 (12)	0.0626 (12)	0.0601 (12)	−0.0020 (10)	−0.0054 (9)	0.0120 (9)
C3	0.0689 (14)	0.0793 (15)	0.0564 (12)	−0.0138 (12)	0.0052 (10)	0.0083 (11)
C4	0.0622 (12)	0.0731 (14)	0.0716 (14)	−0.0145 (11)	0.0189 (11)	−0.0118 (11)
C5	0.0596 (12)	0.0514 (11)	0.0768 (14)	−0.0064 (9)	0.0183 (10)	−0.0085 (10)
C6	0.0478 (9)	0.0437 (9)	0.0596 (11)	−0.0064 (8)	0.0075 (8)	−0.0032 (8)
C7	0.0410 (8)	0.0382 (8)	0.0551 (10)	0.0016 (7)	−0.0019 (7)	−0.0037 (7)
C8	0.0435 (9)	0.0503 (10)	0.0526 (10)	0.0070 (8)	−0.0115 (8)	−0.0111 (8)
C9	0.0520 (10)	0.0376 (8)	0.0588 (10)	−0.0089 (8)	−0.0089 (8)	0.0002 (8)
C10	0.0686 (12)	0.0525 (11)	0.0602 (11)	−0.0076 (10)	−0.0137 (10)	0.0056 (9)
C11	0.0776 (14)	0.0571 (12)	0.0571 (12)	−0.0192 (11)	−0.0018 (10)	0.0028 (9)
C12	0.0769 (15)	0.0618 (12)	0.0669 (13)	−0.0092 (11)	0.0149 (11)	0.0024 (10)
C13	0.0686 (13)	0.0658 (13)	0.0748 (14)	0.0097 (11)	0.0105 (11)	0.0092 (11)
C14	0.0559 (11)	0.0475 (10)	0.0600 (11)	−0.0040 (8)	−0.0032 (9)	0.0054 (8)
C15	0.0543 (10)	0.0355 (8)	0.0583 (10)	−0.0008 (7)	−0.0134 (8)	−0.0021 (7)
C16	0.0625 (11)	0.0325 (8)	0.0637 (11)	−0.0023 (8)	−0.0099 (9)	−0.0051 (8)

### Geometric parameters (Å, °)

**Table:**

S1—C6	1.7285 (19)	N8—H81	0.860 (10)
S1—C7	1.7555 (17)	N8—H82	0.861 (10)
S2—C14	1.733 (2)	C1—C2	1.390 (3)
S2—C15	1.7562 (19)	C1—C6	1.401 (3)
N1—C7	1.305 (2)	C2—C3	1.372 (3)
N1—C1	1.387 (2)	C2—H2	0.9300
N2—C8	1.335 (2)	C3—C4	1.383 (3)
N2—C7	1.360 (2)	C3—H3	0.9300
N3—C8	1.328 (3)	C4—C5	1.375 (3)
N3—H31	0.863 (9)	C4—H4	0.9300
N3—H32	0.860 (10)	C5—C6	1.389 (3)
N4—C8	1.339 (3)	C5—H5	0.9300
N4—H41	0.855 (10)	C9—C10	1.396 (3)
N4—H42	0.857 (10)	C9—C14	1.396 (3)
N5—C15	1.304 (2)	C10—C11	1.368 (3)
N5—C9	1.390 (3)	C10—H10	0.9300
N6—C16	1.321 (3)	C11—C12	1.382 (3)
N6—C15	1.362 (2)	C11—H11	0.9300
N7—C16	1.336 (3)	C12—C13	1.374 (3)
N7—H71	0.862 (10)	C12—H12	0.9300
N7—H72	0.856 (10)	C13—C14	1.388 (3)
N8—C16	1.347 (3)	C13—H13	0.9300
			
C6—S1—C7	89.43 (9)	C5—C6—C1	121.36 (18)
C14—S2—C15	89.54 (9)	C5—C6—S1	129.38 (16)
C7—N1—C1	110.79 (15)	C1—C6—S1	109.25 (14)
C8—N2—C7	119.96 (16)	N1—C7—N2	130.40 (16)
C8—N3—H31	117.0 (16)	N1—C7—S1	115.11 (13)
C8—N3—H32	121.0 (16)	N2—C7—S1	114.48 (12)
H31—N3—H32	122 (2)	N3—C8—N2	124.73 (18)
C8—N4—H41	116.5 (19)	N3—C8—N4	118.86 (18)
C8—N4—H42	118.1 (17)	N2—C8—N4	116.41 (18)
H41—N4—H42	125 (2)	N5—C9—C10	125.84 (18)
C15—N5—C9	111.18 (16)	N5—C9—C14	115.27 (17)
C16—N6—C15	120.07 (16)	C10—C9—C14	118.89 (19)
C16—N7—H71	115.0 (18)	C11—C10—C9	119.3 (2)
C16—N7—H72	118.9 (17)	C11—C10—H10	120.3
H71—N7—H72	125 (2)	C9—C10—H10	120.3
C16—N8—H81	117.6 (16)	C10—C11—C12	121.4 (2)
C16—N8—H82	119.8 (17)	C10—C11—H11	119.3
H81—N8—H82	116 (2)	C12—C11—H11	119.3
N1—C1—C2	125.81 (18)	C13—C12—C11	120.6 (2)
N1—C1—C6	115.41 (16)	C13—C12—H12	119.7
C2—C1—C6	118.76 (18)	C11—C12—H12	119.7
C3—C2—C1	119.6 (2)	C12—C13—C14	118.5 (2)
C3—C2—H2	120.2	C12—C13—H13	120.7
C1—C2—H2	120.2	C14—C13—H13	120.7
C2—C3—C4	121.1 (2)	C13—C14—C9	121.3 (2)
C2—C3—H3	119.5	C13—C14—S2	129.41 (17)
C4—C3—H3	119.5	C9—C14—S2	109.25 (15)
C5—C4—C3	120.6 (2)	N5—C15—N6	130.46 (18)
C5—C4—H4	119.7	N5—C15—S2	114.76 (15)
C3—C4—H4	119.7	N6—C15—S2	114.79 (13)
C4—C5—C6	118.5 (2)	N6—C16—N7	124.9 (2)
C4—C5—H5	120.7	N6—C16—N8	116.38 (18)
C6—C5—H5	120.7	N7—C16—N8	118.7 (2)
			
C7—N1—C1—C2	178.46 (18)	C15—N5—C9—C10	−178.95 (18)
C7—N1—C1—C6	0.1 (2)	C15—N5—C9—C14	0.4 (2)
N1—C1—C2—C3	−178.35 (19)	N5—C9—C10—C11	−179.80 (18)
C6—C1—C2—C3	−0.1 (3)	C14—C9—C10—C11	0.9 (3)
C1—C2—C3—C4	0.4 (3)	C9—C10—C11—C12	−0.6 (3)
C2—C3—C4—C5	−0.2 (4)	C10—C11—C12—C13	−0.1 (3)
C3—C4—C5—C6	−0.2 (3)	C11—C12—C13—C14	0.5 (4)
C4—C5—C6—C1	0.5 (3)	C12—C13—C14—C9	−0.1 (3)
C4—C5—C6—S1	178.91 (17)	C12—C13—C14—S2	179.90 (18)
N1—C1—C6—C5	178.08 (17)	N5—C9—C14—C13	−179.97 (19)
C2—C1—C6—C5	−0.4 (3)	C10—C9—C14—C13	−0.6 (3)
N1—C1—C6—S1	−0.6 (2)	N5—C9—C14—S2	0.0 (2)
C2—C1—C6—S1	−179.04 (15)	C10—C9—C14—S2	179.40 (14)
C7—S1—C6—C5	−177.9 (2)	C15—S2—C14—C13	179.7 (2)
C7—S1—C6—C1	0.65 (14)	C15—S2—C14—C9	−0.30 (14)
C1—N1—C7—N2	179.79 (18)	C9—N5—C15—N6	179.41 (18)
C1—N1—C7—S1	0.39 (19)	C9—N5—C15—S2	−0.6 (2)
C8—N2—C7—N1	1.6 (3)	C16—N6—C15—N5	19.2 (3)
C8—N2—C7—S1	−178.99 (13)	C16—N6—C15—S2	−160.82 (14)
C6—S1—C7—N1	−0.63 (15)	C14—S2—C15—N5	0.53 (15)
C6—S1—C7—N2	179.88 (14)	C14—S2—C15—N6	−179.47 (15)
C7—N2—C8—N3	−1.5 (3)	C15—N6—C16—N7	−5.5 (3)
C7—N2—C8—N4	177.80 (17)	C15—N6—C16—N8	176.74 (17)

### Hydrogen-bond geometry (Å, °)

**Table:**

*D*—H···*A*	*D*—H	H···*A*	*D*···*A*	*D*—H···*A*
N3—H31···N1	0.86 (1)	2.00 (2)	2.679 (2)	134 (2)
N3—H32···N2^i^	0.86 (1)	2.40 (2)	3.228 (2)	161 (2)
N4—H41···N6	0.86 (1)	2.25 (1)	3.084 (3)	165 (3)
N4—H42···N8^i^	0.86 (1)	2.50 (2)	3.350 (3)	176 (2)
N7—H71···N5	0.86 (1)	2.03 (2)	2.717 (3)	136 (2)
N8—H81···N2	0.86 (1)	2.24 (1)	3.096 (3)	177 (2)

Symmetry codes: (i) −*x*+3/2, *y*−1/2, *z*.
